# p55PIK Transcriptionally Activated by MZF1 Promotes Colorectal Cancer Cell Proliferation

**DOI:** 10.1155/2013/868131

**Published:** 2012-12-23

**Authors:** Yu Deng, Jing Wang, Guihua Wang, Yuan Jin, Xuelai Luo, Xianmin Xia, Jianping Gong, Junbo Hu

**Affiliations:** ^1^Molecular Medical Center, Tongji Hospital, Huazhong University of Science and Technology, Wuhan 430030, China; ^2^Department of Surgery, Tongji Hospital, Huazhong University of Science and Technology, Wuhan 430030, China; ^3^Department of Immunology, Tongji Medical College, Huazhong University of Science and Technology, Wuhan 430030, China

## Abstract

p55PIK, regulatory subunit of class IA phosphatidylinositol 3-kinase (PI3K), plays a crucial role in cell cycle progression by interaction with tumor repressor retinoblastoma (Rb) protein. A recent study showed that Rb protein can localize to the mitochondria in proliferative cells. Aberrant p55PIK expression may contribute to mitochondrial dysfunction in cancer progression. To reveal the mechanisms of p55PIK transcriptional regulation, the p55PIK promoter characteristics were analyzed. The data show that myeloid zinc finger 1, MZF1, is necessary for p55PIK gene transcription activation. ChIP (Chromatin immuno-precipitation) assay shows that MZF1 binds to the cis-element “TGGGGA” in p55PIK promoter. In MZF1 overexpressed cells, the promoter activity, expression of p55PIK, and cell proliferation rate were observed to be significantly enhanced. Whereas in MZF1-silenced cells, the promoter activity and expression of p55PIK and cell proliferation level was statistically decreased. In CRC tissues, MZF1 and p55PIK mRNA expression were increased (*P* = 0.046, *P* = 0.047, resp.). A strong positive correlation (*Rs* = 0.94) between MZF1 and p55PIK mRNA expression was observed. Taken together, we concluded that p55PIK is transcriptionally activated by MZF1, resulting in increased proliferation of colorectal cancer cells.

## 1. Introduction

Activation of the phosphatidylinositol 3-kinase (PI3K)/AKT pathway is thought to play a crucial role in the development of a variety of human cancers. Several academic efforts are underway to define therapeutic inhibitors of the pathway components [1, 2]. PI3K interacts with phosphatidylinositol-3-phosphate at the cell membrane and catalyzes the phosphorylation of downstream effector(s) such as Akt [1]. Class IA PI3Ks, consisting of a catalytic subunits p110 and regulatory subunit (p85, p55, and p50), play a critical role in cell proliferation and cell survival [3–6].

The p55PIK, also known as p55*γ*, is encoded by the pik3r3 gene [7, 8]. We previously reported that p55PIK, the N-terminal 24-amino-acid of which is associated with tumor suppressor retinoblastoma protein (Rb), may play an important role in cell cycle control [9]. Ectopic expression of N-terminal 24-amino-acid of p55PIK inhibited cell cycle progression in several cell lines, such as colorectal (HT29) and thyroid (FTC236) cancer cells [10]. One study reported that elevated p55PIK mRNA expression was observed in ovarian, liver, prostate, and breast cancers. A recent study showed that Rb protein can localize to the mitochondria in proliferative cells [11]. Aberrant p55PIK expression may contributes to mitochondrial dysfunction in cancer progression. In addition, apoptosis was observed in p55PIK downregulated ovarian cancer cell lines [12]. Further more, the insulin-like growth factor 2 (IGF2)-p55PIK interaction involved in promoting the growth of a subset of proliferative glioblastomas that lack EGF receptor amplification [13]. These findings suggest that p55PIK was aberrantly expressed in several human cancers and p55PIK may act as an important target for cancer treatment. The detailed mechanism, especially its transcriptional regulation mechanism, remains unknown. Disclosure of the factor(s) that contribute to the regulation of p55PIK expression may be useful in cancer treatment targeting p55PIK. 

 Several factor(s) have been reported to regulate p55PIK expression in different human disease models, such as mammary cancer [14] and cerebral ischemia-reperfusion [15]. One study has shown that p55PIK expression increased in the presence of doxorubicin, an anthracycline antibiotic that is used abroad in cancer chemotherapy, in breast cancer MDA-MB-231 cells but not in MCF-7 cells [14]. The genetic factors altered in MDA-MB-231 cells, which are p53/estrogen receptor/progesterone receptor negative, may be involved in regulation of p55PIK expression. Another study reported that the Insulin-like growth factor 2 (IGF2) and p55PIK are overexpressed in more proliferative glioblastomas [13]. In fatty acid and cholesterol biosynthesis, Sterol-regulatory element binding protein-1 (REBP-1) and Platelet-derived growth factor (PDGF) induce the expression of p55PIK in AG01518 human foreskin fibroblasts [16]. A recent study revealed that berberine, an effective candidate neuroprotective agent in clinical ischemic stroke, enhances p55PIK promoter activity during cerebral ischemia-reperfusion [15]. In *Mycobacterium tuberculosis* model of WI-38 cells, downregulated p55PIK expression was observed by recombinant* Mycobacterium tuberculosis* CFP-10/ESAT-6 protein treatment [17]. Despite the clarification of these factors, little is known about the mechanism of p55PIK transcriptional regulation.

The aim of the present study is to identify the cis-elements and transcription factor(s) involved in p55PIK transcriptional activation in colorectal cancer cells (CRCs). Firstly, we made *in silico* analysis and deletion analysis of the p55PIK gene promoter and determined the transcriptional factor(s) that may regulate p55PIK transcription. We also evaluated the influence of the transcriptional factors(s) on PI3K expression and the cell growth of CRC cells. Based on the results of this study, the transcription factor(s)-p55PIK axis may be suggested as the potentially crucial target(s) of CRC treatment.

## 2. Materials and Methods

### 2.1. Ethics Statement

All research involving human participants has been approved by the Huazhong University of Science and Technology Ethics committee. We obtained informed, written consent from all participants involved in this study.

### 2.2. Cell Culture and Transfection

Cell lines HepG2, HeLa, SW480, and LoVo were purchased from the American Type Culture Collection (Manassas, VA, USA) and cultured in DMEM supplemented with 10% fetal bovine serum (HyClone, Logan, UT, USA). These cell lines were cultured at 37°C in 5% CO_2_/air atmosphere. Transfection was done using Lipofectamine 2000 (Invitrogen, Carlsbad, CA, USA) following the manufacturer's instructions.

### 2.3. Reporter Constructs and Expression Vectors

DNA fragments containing the same 3′ terminal and different 5′ terminal of p55PIK promoter (Table 1) were amplified by PCR from human genomic DNA and cloned into pGL3 Basic vectors (Promega, Madison, WI, USA) between kpn1 and Bgl II restriction enzyme sites. Reconstructed reporter plasmids were named as (−1633/+45)-p55PIK, (−1243/+45)-p55PIK, (−1064/+45)-p55PIK, (−839/+45)-p55PIK, or (−651/+45)-p55PIK), respectively. Before use, all constructs were verified as correct by sequencing. MZF1 expression vector MZF1-GFP and GFP-control vector were kindly provided by Zhou et al. [18].

### 2.4. Small Interfering RNA

Synthetic siRNA targeting human MZF1 (RuiBo, Guangzhou, China) was transfected into cultured cells. Transfection was done using Lipofectamine 2000 following manufacturer's instructions. Cells were cultured in 24-well plates in antibiotic-free 10% fetal bovine serum plus medium and transfected with 50 nmol/L siRNA at 70–80% confluency. Expression of MZF1 or p55PIK was detected at 24 h or 48 h after-transfection.

### 2.5. Site-Directed Mutagenesis

Constructs bearing mutant promoter variants of p55PIK were generated by PCR using the wildtype p55PIK reporter construct (−1243/+45)-p55PIK as template. Underlined nucleotides in Table 2 indicate mutated sequences. Primers were designed according to manufacturer's instructions and produced by Invitrogen. Site-directed mutagenesis was done according to manufacturer's protocol for the Quick Change site-directed mutagenesis kit (Stratagene, La Jolla, CA, USA). All mutants of (−1243/+45)-p55PIK were verified correctly by sequencing.

### 2.6. Dual-Luciferase Reporter Gene Assay

Cells were seeded in 24-well plates. After culturing for 24 h, cells were cotransfected with luciferase reporter plasmids and *Renilla* vector (pRL-TK) (Promega, Madison, WI, U.S.A). Luciferase activities were measured at 24 h post-transfection, using the Dual-Luciferase Reporter Assay System (Promega, Madison, WI, U.S.A). Luciferase activity was normalized for transfection efficiency using the corresponding *Renilla* luciferase activity. All experiments were performed independently at least four times.

### 2.7. Chromatin Immunoprecipitation (ChIP) Assay 

The chromatin immunoprecipitation (ChIP) assay was used to show interactions between transcription factor(s) and p55PIK promoter DNA sequence in CRC cell lines or tissues samples. In brief, chromatin from SW480 cells or CRC tissues was sheared to DNA fragments with an average size of 200–500 bp. After cross-linking reversal and proteinase-K digestion, each individual IP was purified using QIA-quick PCR purification kit (Qiagen, Valencia, CA, USA) followed by elution with 50 *μ*L of elution buffer. After elution, IPs were amplified using PCR. To detect the transcription factor(s) binding motif in the p55PIK promoter, we used sense primer 5′-GAAGCCTAGAGAGCGGT-3′ and antisense primer 5′-TGTCAAGTGCCTGAGAAC-3′. MZF1 antibody (Santa Cruz, CA, USA) and control IgG (Santa Cruz, CA, USA) were used to immunoprecipitate the protein-DNA complex.

### 2.8. Real-Time PCR

The comparative Ct method with SYBR Green was conducted with the ABI 7300 Real-Time PCR System (Applied Biosystems, Foster City, CA, USAs). Forp55PIKdetection, the following primers were used: sense primer 5′-GAGTATGGACCGCGATGA-3′, antisense primer 5′-TTGGCTTAGGTGGCTTTG-3′; MZF1: sense primer 5′-AGTGTAAGCCCTCACCTCC-3′, antisense primer 5′-GGGTCCTGTTCACTCCTCAG-3′; GAPDH sense primer: 5-AACGGATTTGGTCGTATTG-3, antisense primer 5-GAAGATGGTGATGGGATT-3.

### 2.9. Western Blot Analysis

Cells were washed in cold PBS and incubated with RIPA buffer (Biomed, Beijing, China) for 30 min at 4°C. Cell lysates were centrifuged, and proteins were collected and separated by gel electrophoresis. The PVDF membranes were blocked in 5% (w/v) milk dissolved with PBS-0.1% Tween 20 (PBS-T) for 1 hr at room temperature. Membranes were then incubated with primary antibodies diluted in PBS-T overnight at 4°C. Membranes were washed with PBS-T and incubated with peroxidase-conjugated secondary antibody diluted in PBS-T for 1 hr at room temperature. Membranes were washed in PBS-T; bound antibody was detected with ECL western blotting detection reagents (Thermo Scientific, Rockford, IL, USA). The primary antibody was anti-MZF1, anti-p55PIK, or anti-GAPDH (all purchased from Santa Cruz, Biotechnology, Santa Cruz, CA, USA), respectively. All experiments were repeated 3 times. Protein bands were quantified using Quantity One software (Bio-Rad).

### 2.10. MTT Assay

For measurements of cell growth, cells were counted manually, plated in triplicate in 96-well plates at 3 × 10^3^ and 1 × 10^4^ cells/well, and transfected with plasmids or SiRNA for 72 h to 96 h; media were then removed and 50 *μ*L of 3-[4,5 dimethylthiazol-2-y]-2,5-diphenyltetrazolium bromide (MTT; Sigma-Aldrich) was added to each well. After incubation for 4h, MTT-containing media were removed, and 100 *μ*L of dimethyl sulfoxide (DMSO) was added to each well. Plates were placed on a plate shaker for 20 mins and the optical density was read immediately at 595 nm/650 nm with a model DTX 880 microplate reader (Beckman Coulter, Fullerton, CA, USA).

### 2.11. Statistics

All measurements were performed in at least three independent experiments. The means ± SD were calculated. Student's *t* test was used to compare two independent groups. For all tests, values of *P *< .05 were considered statistically significant.

## 3. Results and Discussion

### 3.1. Deletion Analysis of the p55PIK Promoter

p55PIK, the regulatory subunit of class IA phosphatidylinositol 3-kinase (PI3K), plays a crucial role in cell cycle progression. We previously reported that p55PIK, the 24-amino-acid N-terminal end (N24) of which is associated with tumor suppressor retinoblastoma protein (Rb), may play an important role in cell cycle control [9]. A recent study showed that Rb protein can localize to the mitochondria in proliferative cells [11]. Aberrant p55PIK expression may contributes to mitochondrial dysfunction in cancer progression. To reveal the mechanisms of p55PIK transcriptional regulation, the p55PIK promoter characteristics were analyzed.

**Figure 1:**
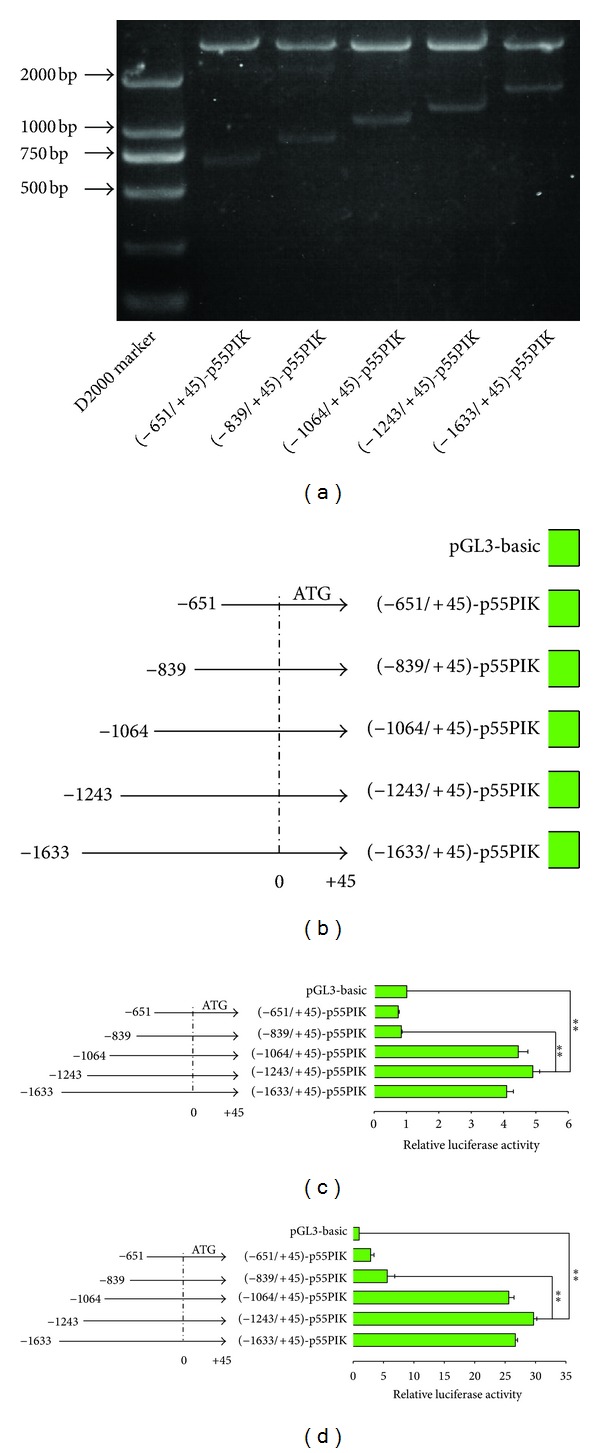
Main p55PIK promoter activity conferred by −1243/−840 upstream region sequence. (a) Series of 5′ end p55PIK promoter recombinant reporter plasmids; (b) DNA fragments with different lengths but with the same 3′ end of p55PIKpromoter cloned into pGL3-Basic reporter plasmids; (c, d) Relative luciferase activity (RLA) of p55PIK reporter plasmids was examined with Dual-Luciferase reporter assay in HepG2 and HeLa cell lines. Data are shown as mean ± SD (*n* = 4; **P < .01, per Student's *t* -test.)

To identify cis-acting elements within the p55PIK promoter, we constructed a series of luciferase reporter plasmids that contained 5′-deletions of the p55PIK promoter at nucleotides (nt) −1633, −1243, −1064, −839, and −651, with a common 3′-terminus at +45 (Figures 1(a), 1(b)). The promoter activities of the 5′-deletions mutants were assessed by Dual-Luciferase Reporter Assay after transient transfection into two human cell lines: cervical carcinoma HeLa and hepatocellular carcinoma HepG2, which have higher expression of p55PIK than other cell lines (data not shown). The constructs (−839/+45)-p55PIK and (−651/+45)-p55PIK showed similar promoter activity compared with pGL3-Basic vector, whereas the (−1633/+45)-p55PIK, (−1243/+45)-p55PIK, and (−1064/+45)-p55PIK showed higher activities compared with control vector (Figures 1(c), 1(d)). Importantly, most remarkable changes of promoter activity were observed between constructs (−1243/+45)-p55PIK and (−839/+45)-p55PIK. These results suggested that p55PIK promoter activity is largely lost upon deletion of the sequence between −1243 and −839 of the full-length promoter.

The first set of experiments was designed to analyze the characteristics of p55PIK promoter, and to identify the most commonly activated promoter region of p55PIK in cancer cells, we performed luciferase-based reporter assays. The data demonstrate that the full length of p55PIK promoter located at −1243/+45 upstream of the translation start site (ATG) and the −1243/−840 region were responsible for p55PIK gene transcription. We mapped the p55PIK promoter to find its most activated fragments. A series of 5′ flanking of p55PIK promoter DNA fragments (−1633/+45, −1243/+45, −1064/+45, −839/+45, and −651/+45) were cloned into luciferase-reporter construct and the relative luciferase activity was measured in cancer cells. The results show that −1243/+45 fragment confers highest promoter activity and −840/+45 fragment presents a significant decrease compared with −1243/+45 fragment. The current study indicate that the cis-elements were located at the −1243/−840 region of p55PIK promoter.

### 3.2. Identification of Cis-Acting Elements Controlling p55PIK Expression

**Figure 2:**
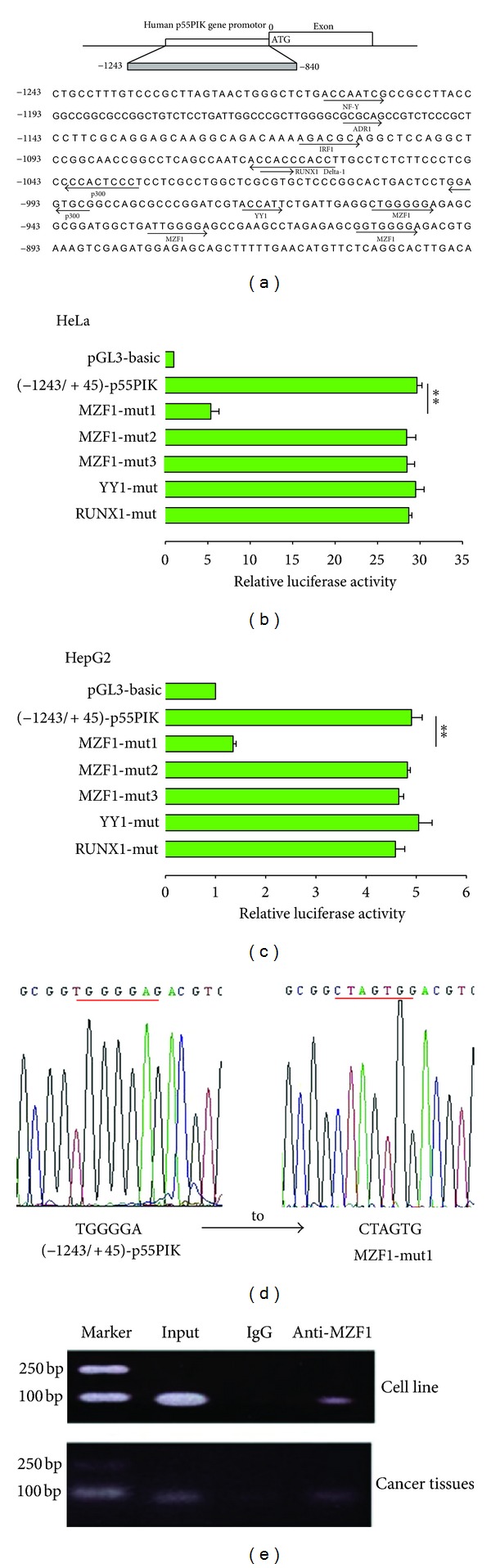
Transcription factor MZF1 binds on site of 5′ end of p55PIK, inducing p55PIK promoter activity. (a) Analysis of the −1243/−840 region, which affects main p55PIK promoter activity and transcription. Several transcription factors, including MZF1, YY1, Runx1, ADR1, IRF1, Delta1, or p300, bind on corresponding domains within p55PIK promoter. (b, c) Relative luciferase activity (RLA) at 24 h after-transfection of a series of mutant constructs of p55PIK reporter plasmids (−1243/+45)-p55PIK, named MZF1-mut1, MZF1-mut2, MZF1-mut3, YY1-mut, and RUNX1-mut are transfected into HepG2 or HeLa cell lines. Data are shown as mean ± SD (*n* = 4; **P < .01, per Student's *t*-test). (Data of ADR1-mut, IRF1-mut, Delta1-mut, p300-mut1, and p300-mut2 are not shown). (d) Sequencing of mutant MZF1-mut1, showing mutagenesis from TGGGGA to CTAGTG at the −901/−896 site, of p55PIK reporter plasmids (−1243/+45)-p55PIK. Sequencing data show that associated binding site was mutated successfully on (−1243/+45)-p55PIK reporter plasmids (data not shown). (e) Transcription factors MZF1 bind to p55PIK promoter region. Chromatin immunoprecipitation (ChIP) assay was used to detect binding between MZF1 protein and TGGGGA sequence located at −901/−896 site of p55PIKpromoter in colon cancer cells and resected tissues.

To identify the critical cis-enhancing elements in the −1243/−840 region, we generated various mutant reporter based on the (−1243/+45)-p55PIK plasmid with substitution mutations by site-directed mutagenesis. As shown in Figure 2(a), the potential transcription factor binding sites of YY1, MZF1, Runx1, ADR1, IRF1, Delta1, and p300 were found in the −1243/−840 region based on motif analysis. The introduction of a TGGGGA site mutation (from TGGGGA to CTAGTG) which located at −901/−896 markedly reduced the luciferase activity of (−1243/+45)-p55PIK (Figure 2(d)), whereas mutation of YY1, RUNX1, or other MZF1 binding sites did not affect the promoter activity of (−1243/+45)-p55PIK (Figures 2(b), 2(c)). These results demonstrated that the putative MZF1 binding site TGGGGA located at −901/−896 region was crucial for functioning of the p55PIK promoter. 

The focus of the second set of experiments was to identify the cis-element(s) of p55PIK promoter and corresponding transcription factor(s). The preliminary sequence analysis of the domain −1243/−840 reveals the presence of several nonoverlapping cis-elements and corresponding transcription factors. It was reported that MZF1 binds to the 5′-AGTGGGGA-3′ or 5′-CGGGnGAGGGGGAA-3′ sequence of gene promoters to regulate the expression of a target gene [19]. YY1, which may bind to the 5′-ACCATTC-3′ site of the p55PIK promoter, is a ubiquitously distributed zinc-finger-type transcription factor, involved in regulating a variety of promoters [16–18]. Runx1, which may bind to the 5′-CACCACCC-3′ sequence of the p55PIK promoter, is essential for hematopoietic development [19, 20]. Interferon regulatory factor 1 (IRF1), which may interact with the 5′-AGACGC-3′ DNA binding site, was initially described as a transcription factor able to activate expression of the cytokine interferon beta. IRF-1 plays important roles in immune response [21, 22], apoptosis [23, 24], and tumor suppression [25]. The p300/CBP coactivator family interacts with transcription factors p53 [26] and STAT3 [27] to transcriptionally activate the expression of their target genes. Only the mutant MZF1-mut1 shows decreased luciferase activity compared with the wildtype promoter plasmids.

### 3.3. MZF1 Binding on the TGGGGA Site Located at −901/−896 Region of the p55PIK Promoter in Colon Cancer Cell and Tissues

Next, to further verify whether MZF1 is involved in p55PIK transcription, we employed primers spanning the putative MZF1-binding site of the p55PIK promoter to perform chromatin immunoprecipitation (ChIP) assays, confirming the presence of endogenous MZF1 bound to this region in CRC cell line SW480 or CRC tissues. In Figure 2(e), the specific sequence within p55PIK promoter was precipitated from cell lysates by anti-MZF1 antibody but not by control IgG in both CRC cell line SW480 and CRC tissues. Thus, the data strongly indicate that MZF1 binds to TGGGGA domain in the proximal promoter of p55PIK in CRC cells or tissues.

### 3.4. p55PIK Is Transcriptionally Activated by MZF1

**Figure 3:**
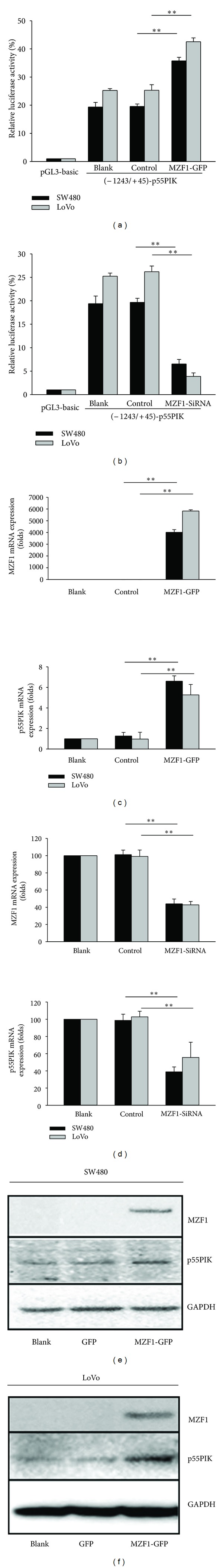
p55PIK expression is transcriptionally activated by MZF1 in human colon cancer cell lines SW480 and LoVo. Cells were transfected with a reporter plasmid for p55PIK promoter (−1243/+45)-p55PIK and cotransfected with or without MZF1-GFP expression plasmids or MZF1 SiRNA for 24 h. (a, b) Relative luciferase activity (RLA) was determined as described under “Materials and Methods”; (c), (d), mRNA expression of MZF1 or p55PIK was detected by real-time PCR; (e), (f) cell lysates were analyzed by MZF1, p55PIK, or GAPDH immunoblot. Data are shown as mean ± SD (*n* = 3; **P < 0.01, per Student's *t*  test).

To assess the role of MZF1 in transcriptional activity of p55PIK, we measured the luciferase activity of the p55PIK promoter construct after transfection of MZF1 expression plasmids or MZF1-SiRNA in CRC cell SW480 and LoVo. Increased luciferase activity was observed in MZF1 overexpressed cells (Figure 3(a)).  Figure 3(b) shows decreased luciferase activity after MZF1 SiRNA transfection. p55PIK mRNA expression was evaluated in MZF1 over-expressing or MZF1-silencing CRC cells. As shown in Figures 3(c) and 3(d), p55PIK mRNA increased after MZF1 was overexpressed and decreased after MZF1 was silenced, respectively. By Western Blot analysis, MZF1 over-expressing CRC cell SW480 and LoVo showed increased p55PIK expression (Figures 3(e), 3(f)). Altogether, these findings indicate that MZF1 functions at least in part as a transcriptional regulator of p55PIK.

### 3.5. Transcriptional Activation of p55PIK by MZF1 Resulting in Accelerated Cell Proliferation in CRC Cell Lines

Myeloid zinc finger 1 (MZF1), a transcription factor belonging to the Krüppel zinc finger protein family, was previously reported as an important factor whose aberrant expression disturbed hematopoietic cell proliferation and cell tumorigenesis [19, 28, 29]. Transcription factor MZF1, binding on the DNA-binding consensus sequence of 5′-AGTGGGGA-3′ or 5′-CGGGnGAGGGGGAA-3′ [19], regulated several genes' expression which play important role in cancer migration, invasion, or cancer differentiation. Human liver cancer cell line treated with MZF1 antisense oligonucleotide showed repressed protein kinase C *α* expression and inhibited subcutaneous tumor growth in nude mice [30]. MZF1 transcriptionally regulates Axl receptor tyrosine kinase gene in human colon cancer or cervical cancer, which induced migration and metastasis of colon cancer *in vitro* and *in vivo* [31]. mRNA expression of MZF1 and AXL, with significant correlation, were both upregulated in colorectal cancer [31]. Raised cell cycling and loss of contact inhibition were detected in MZF-1 overexpressed NIH 3T3 cells [19]. Therefore, MZF1 may function as an oncogene in solid cancer.

**Figure 4:**
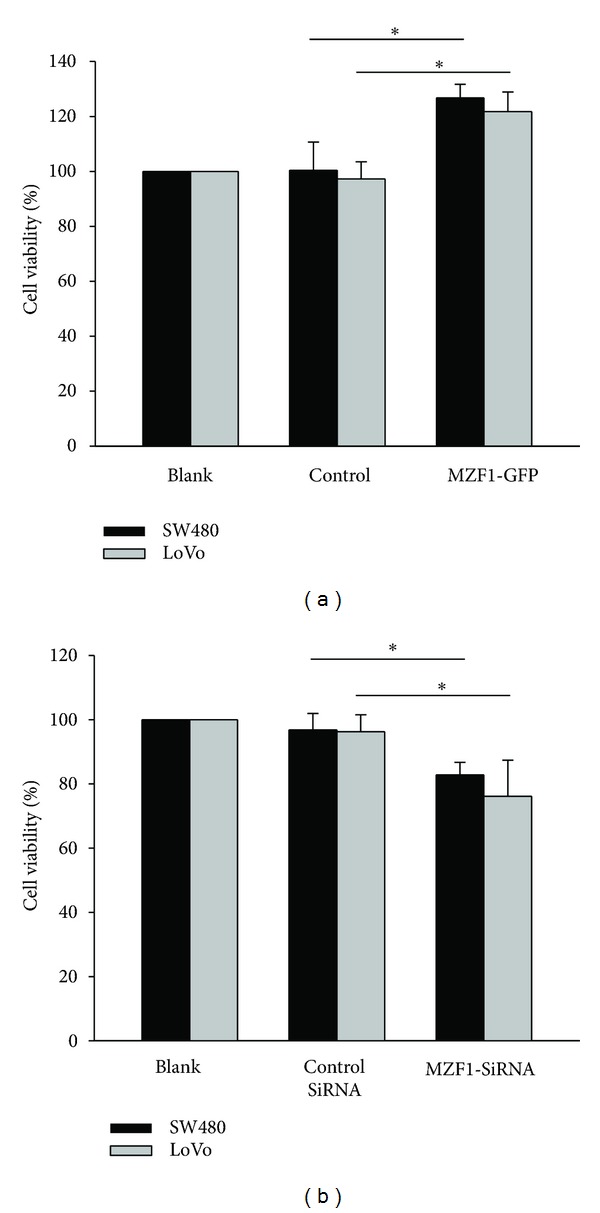
Transcriptional activation of p55PIK by MZF1 protein gives rise to cancer cell growth. Cells were transfected with a reporter plasmid for p55PIK promoter (−1243/+45)-p55PIK and cotransfected with or without MZF1-GFP expression plasmids or MZF1 SiRNA for 72 h to 96 h (a, b) Cell viability was detected with MTT assay as described under “Materials and Methods.”  Data are shown as mean ± SD (*n* = 3; *P < 0.05, per Student's *t*  test).

Next, to determine the effects of the MZF1 induced p55PIK transcriptional activation on the growth, the CRC cell lines were examined with the MTT assay. We found that MZF1-GFP could induce acceleration of the proliferation of CRC (Figure 4(a)) and that MZF1-SiRNA could induce inhibition of growth of CRC (Figure 4(b)) as well.

### 3.6. Relationship between Expression of MZF1 and p55PIK in CRC Tissues

**Figure 5:**
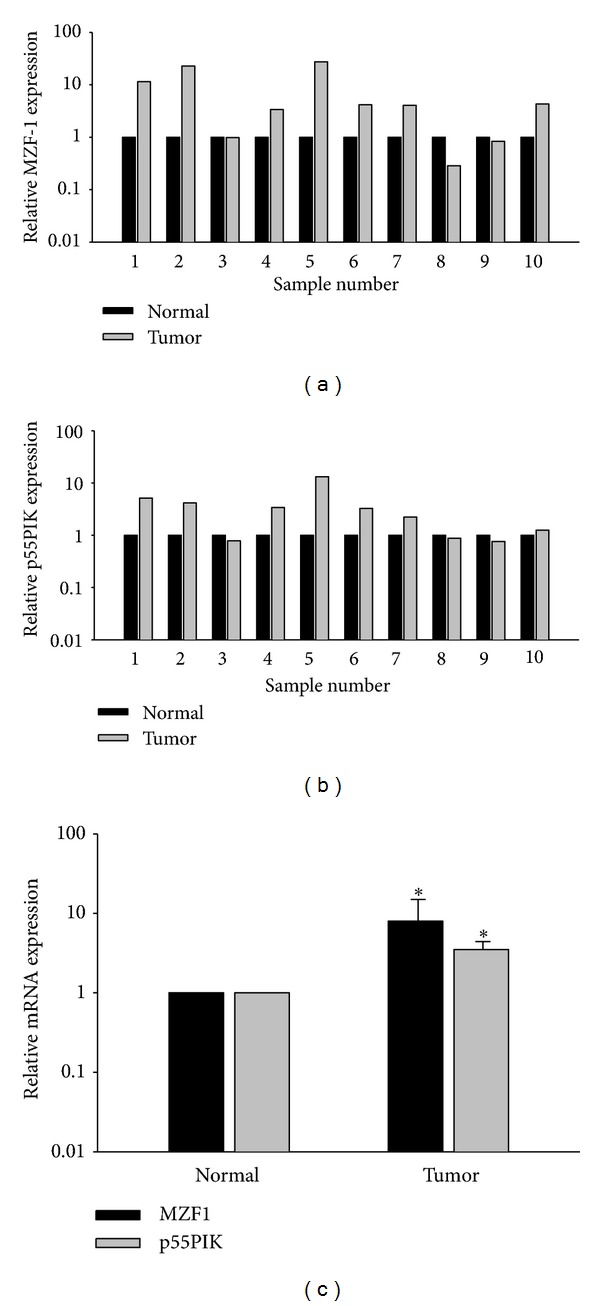
MZF1 and p55PIK expression are significant correlated in resected colorectal tissues.Total RNA was extracted from fresh-frozen human colorectal cancer samples. (a, b) Real-time PCR results comparing MZF1 and p55PIK expression in tumors and corresponding normal tissues. (c) Expression of MZF1 and p55PIK in normal tissues were normalized, and differences between normal and tumor tissues in all 10 patients were analyzed for average MZF1 or p55PIK expression (*P ≤ .05, per Student's *t* -test).

Finally, to demonstrate the relationship between MZF1 and p55PIK expression in human CRC, we examined endogenous expression of MZF1 and p55PIK in 10 resected CRC tissue samples and corresponding normal mucosal tissues from the same patient received therapeutic surgery. Tumors from 7 of 10 patients showed significant increased expression of MZF1 and p55PIK compared with normal tissues (*P* = 0.046 and *P* = 0.047, resp.); MZF1 gene expression was positively and significant correlated with p55PIK expression in the resected tumor tissues (*Rs* = 0.94; *P* < .05), indicating that MZF1 and p55PIK are involved in tumorigenesis (Figure 5).

## 4. Conclusion

In summary, we have shown that the transcription factor MZF1, which directly binds to its cis-element within the p55PIK promoter, activates p55PIK expression and acts as a growth accelerator in CRC cells. We also demonstrate that the expression of MZF1 and p55PIK is significant correlated, and they are both overexpressed in resected CRC tissues. This investigation may suggest a strategy for development of therapies on p55PIK-associated cancer especially mitochondrion associated cancer.

## Figures and Tables

**Table 1:** Primers for reporter gene constructs of p55PIK.

Primer name	Sequence
pGL3-p55PIK-(−651/+45) up	5′-TTAATTGGTACCGGTCGGGTTGGTTCTTAC-3′
pGL3-p55PIK-(−839/+45) up	5′-TTAATTGGTACCGAATCCCGGTACATCG-3′
pGL3-p55PIK-(−1064/+45) up	5′-TTAATTGGTACCCACCCACCTTGCCTCT-3′
pGL3-p55PIK-(−1243/+45) up	5′-TTAATTGGTACCCTGCCTTTGTCCCGCTTA-3′
pGL3-p55PIK-(−1633/+45) up	5′-TTAATTGGTACCAGGTGGCAAGTGGGTT-3′
pGL3-p55PIK-shared down	5′-ATATATAGATCTCCAGTCTGCGTCATCG-3′

**Table 2:** Primers for site-directed mutagenesis of construct (−1243/+45) p55PIK.

Name	Sequence change	Sense primer sequence (5′-3′)/antisense primer sequence (5′-3′)
RUNX1-mut	ACCACC to GTGTAT	GCAACCGGCCTCAGCCAATCGTGTATCACCTTGCCTCTCTTCCCTC
		GAGGGAAGAGAGGCAAGGTGATACACGATTGGCTGAGGCCGGTTGC
MZF1-mut1	TGGGGA to CTAGTG	GAAGCCTAGAGAGCGGCTAGTGGACGTGAAAGTCGAG
		CTCGACTTTCACGTCCACTAGCCGCTCTCTAGGCTTC
MZF1-mut2	GGGGG to TAGTC	CGTACCATTCTGATTGAGGCTTAGTCAGAGCGCGGATGGCTGATTGGG
		CCCAATCAGCCATCCGCGCTCTGACTAAGCCTCAATCAGAATGGTACG
MZF1-mut3	TGGGGA to CTAGTG	CGAGAGGCGTGGCTGATCTAGTGGCCGAAGCCTAGAGAGCGG
		CCGCTCTCTAGGCTTCGGCCACTAGATCAGCCACGCCTCTCG
YY1-mut	ACCAT to GTTGA	CCAGCGCCCGGATCGTGTTGATCTGATTGAGGCTGG
		CCAGCCTCAATCAGATCAACACGATCCGGGCGCTGG
